# The Independent Practitioner in New York

**Published:** 1881-09

**Authors:** 


THE INDEPENDENT PRACTITIONER IN NEW YORK CITY.

